# President’s Address1Read at the Thirty-ninth Annual Meeting of the American Veterinary Medical Association.

**Published:** 1902-09

**Authors:** J. F. Winchester

**Affiliations:** Lawrence, Mass.


// THE JOURNAL

OF

COMPARATIVE MEDICINE AND VETERINARY ARCHIVES.

VOL. XXIII. *SEPTEMBER, 1902.* No. 9.

### PRESIDENT'S ADDRESS.[1]

BY J. F. WINCHESTER, D.V.S.,
LAWRENCE, MASS.

IT is fitting that I should first express to you my deep sense of appreciation of the compliment and honor you conferred upon me when, by unanimous vote, you elected me to preside over this body. Fully realizing that it is the highest honor that can be bestowed upon an individual member, I assure you that I shall do my best to fulfil the duties of my high office as befits one who has the welfare of the profession very closely at heart.

The present and future success of this Association depends upon the members individually and collectively. We are numerically the largest association of the kind in the world, and it becomes the duty of the members to do their utmost to make the American Veterinary Medical Association the most influential in work and wisdom.

It is now twoscore years since the first gathering of veterinarians was held on this continent That space of time marks an epoch in the life of man when he is ordinarily at the zenith of vigor of mind and body. It is not so with this Association, for its influence will steadily increase, growing grander and more glorious as the years pass by. Its prestige to-day is well shown in that the invitation to hold the meeting in this State was the most cordial one ever extended to it.

The veterinarians of Minnesota are to be congratulated upon having the Governor, the State Board of Health, the Mayor of

[1] Read at the Thirty-ninth Annual Meeting of the American Veterinary Medical Association.

this city, and the Commercial Club so much interested in our profession as to extend individually to this organization the right-hand of fellowship. In behalf of the American Veterinary Medical Association I now thank the gentlemen representing the State and city governments for the courteous treatment that has been offered us.

This Association was organized by about forty veterinarians in 1863, the year following the meeting held in Philadelphia, under the name of the United States Veterinary Medical Association. Since that time the scope of the work has so enlarged that the present name was adopted as indicative of the growth of membership. Through its encouragement and support a veterinary periodical was first published; through its influence the course of study of veterinary medicine in most colleges has been increased to three years, and it is to be hoped that the same influence will cause the few two-year schools that remain to lengthen their period of instruction. There are to-day thirteen colleges graduating veterinarians eligible to membership in this Association, while only five remain that do not comply with our requirements.

As it is my opinion that the requirements of Article I., Chapter VI., of our By-Laws have been instrumental in increasing the period of instruction in many colleges, I offer these suggestions: First, that all alumni from colleges now requiring three years' attendance be eligible for membership in this Association; second, that the graduates from colleges that within two years adopt a three-years' course and otherwise comply with our requirements be eligible to membership.

As there are at the present time only five colleges having at least four veterinarians on their teaching staff that do not require the three-years' course of six months' attendance or more, I trust that the day is not far distant when the term for graduation for all colleges will be extended to four years. If it were a fact that all of our three-year colleges were insisting on like qualifications for matriculation and graduation, with the anticipation of a four years' course in the near future, the confidence of the public would be gained.

This condition of affairs can be brought about by having the trustees of each college appoint delegates with power to act in the matter. The next step in order would be the selection of men of broad and liberal education, who are specialists in the subjects taught, to constitute the faculties of these schools.

This once accomplished, the requirements for matriculation of students in all the colleges being made equal, the results will be that in the near future all cities will have a veterinary health officer acting with the medical officers of health in controlling and preventing diseases; the public will cease to look upon the veterinarians as mere animal physicians and surgeons, and will grant them the privilege of interpreteting and administering laws relating to public health. We should use our moral influence to bring this to pass, that every graduated veterinarian may some time have the prestige due him.

The growth of this Association and interest in it are due to a great extent to its Secretary. His duties are many and arduous, but how few of us have for once stopped to consider them. On his untiring zeal and his ever anticipating the wants of this organization depend the success of our meetings and the interest taken in them.

In looking over the list of Secretaries you will find that, with one exception, our present official has served us the longest; but bear in mind that, with that exception from 1880 to 1888, the duties were very slight compared with those of to-day.

During the first years the membership was limited. Notices of meetings were given in the *Review* and the JOURNAL, and the proceedings of the meetings were not published as at present. The time occupied was one day in September and March, New York City being usually the Mecca in September, and Boston in March. There were but few papers read, the time being principally occupied in changing the By-Laws and discussing contagious pleuropneumonia.

Those of us who were members in the early days little thought that such a change would take place in this Association, for, as I have said, it is to-day the largest numerically in the world. The work having increased to such dimensions, our Secretary should have under his direction as State Secretaries men who are earnest and willing to serve. Before making appointments it would, in my judgment, be desirable to obtain from each man an assurance of his willingness to accept the office and assist in the work which properly belongs to it. Since the duties of the State Secretaries are not defined in our By-Laws, the Secretary should be provided with printed instructions, such as the Executive Committee may recommend, which he may send to the State Secretaries when notifying them of their appointment.

The suggestion has been offered that an effort be made to have the various State Associations elect as members only such as are eligible to membership in this body, and then to have these various organizations endeavor to interest their members, that all may become associated with us. I deem this advisable, as in this way our membership would be increased and our influence greatly extended. Should the State Associations carry out this suggestion, how many new students would the two-year colleges matriculate?

It is now several years since a Prize Committee has been in existence, but I trust that before long we may again see the formation of that committee.

It cannot be said that original work is not being done on this continent. The interesting and valuable papers that are presented not only at our meetings, but also at State Associations, deserve more than a passing notice. Knowing what literary work our members have done without the hope of pecuniary gain, but simply from the desire to aid their profession, would not a prize offered by the Association serve to stimulate for the future those men who have already aided the Association and profession, and at the same time be an incentive to others to interest themselves in such original work? While it is a fact that our profession offers opportunities second to none for the investigator, at the same time are there not a great many of us taking conditions for granted that by the acts of nature resolve themselves without our knowledge of the cause?

In order to gain in wisdom and to benefit the country the name of which we have assumed, what reason can there be why we should neglect to use our influence to induce our members to give to the world the results of their investigations? Let this Association decide on some method, so that it may put its stamp of approval upon the work of its members.

You have caused the formation of a Committee on the Pharmacopœia, the results of their labors to be the official work of this Association, and your President having made a very careful canvas of the membership, to obtain the most eligible men to comprise the committee, there is reason to believe that a body has been gotten together that will do its work thoroughly and reflect credit upon the Association.

The proposition that a committee be formed to formulate a standard of soundness of horses is good. In addition I would suggest that a scale be made by which the excellence of an animal for service may be scored. Ordinarily, the services of a veterinarian at exhibitions have been used in regard to soundness only, and his judgment has not been called for when scoring points of excellence.

The attempt to get a special act of incorporation through Congress, which resulted in failure, was made several years ago. It is now time to take up this question again, and if the consensus of opinion does not favor again trying in Washington it may be well for the Association to apply for incorporation under some State law.

In the past few years the mortality and prevalence of infectious and contagious demic and zootic diseases have diminished. The reason for this is that the causes of the various disorders common to man and animal have been investigated and proven. It is well known that the causes of those diseases communicable to man vary in size from micro-organisms to parasites (if you will allow the comparison), and many of the former do not necessarily belong to the living animal, being found in decaying tissue as well as in storehouses.

Recently the etiology of certain diseases that cause enormous loss in cattle has been demonstrated, and the immunizing of cattle has become a source of wealth to the nation. In this country, so far as I know, those insects which fly at night have not as yet been proven to convey diseases from one animal to another, though they do from man to man; nevertheless, it is known that flies become an active means of contagion among animals.

Tuberculosis, so well known to us all and about which so much controversy has arisen, illustrates, to my mind, the best instance of a malady where hygiene and sanitation are the important factors in its prevention and treatment.

What seems to be the future work of the veterinarian is the prevention of contagious and infectious diseases. As in the past the success which has been attained has been due in a large measure to sanitation, so must we look to that for future success. I would recommend that the Committee on Resolutions draw up an article on the practical value of hygiene. Such a resolution will undoubtedly receive the unqualified approval of the press.

In several of the States important laws have been recently passed which should greatly benefit our profession. I will refer to only one. Within thirty days the veterinarians of New Jersey were fortunate in having a law enacted regulating the practice in that State. This subject will be considered in full by the Committee on Intelligence and Education.

The profession protests against an injustice to a conscientious member on account of the profession, but who is to enter a protest against the veterinarian without personal integrity? There seem to be veterinarians ready and willing to lend themselves to schemes which will further their personal ends. Opportunism seems to be the guide for a great many, but my observation has been that such individuals are successful for awhile only, for in the end those who make use of them turn against them, and not only distrust them, but have not the best opinion of the veterinarian.

These conditions, which are patent to a great many, will, in my judgment, continue to exist if the cause for them is not removed. The lack of a broad education and of self-sacrifice are the evils which this Association should use its influence to eliminate. Morally the remedy is at our door, and a broad, liberal education with self-sacrifice should be strenuously advocated. The beginning must be made when the applicant first raps at the door of our educational institutions, and then continued by having teachers of the required calibre.

A period of instruction covering two, three, or four years cannot offset this evil if the students do not always have before them the example set by broad-minded, self-sacrificing men of culture with whom they are brought in daily contact. Let it be borne in mind that the action of the opportunist is far reaching, and that his seeming opportunity reflects on the profession to such a degree that it will take a long time to correct the wrong done by him.

Throughout the world among the liberal professions there exists a feeling of community and of support. When injustice of any kind strikes one individual member, the entire profession protests, regardless of any other sentiments. They protest against the wrong done, not because of the individual, but on account of the profession of which he is a member. Aside from this professional sentiment, the thought of material support has been overlooked in America by the philanthropists of our profession. It is well manifested by the veterinarians of England and France and by the medical profession of New York State, being expressed by the support and assistance which the members render each other and their families when death or disability overtakes them.

England's National Veterinary Benevolent and Mutual Defence Society has a membership of 250. It was organized to protect members of the veterinary profession against alleged depreciation of animals as a result of sickness, accidents, or opinions honestly expresed in the discharge of their duty; and also to assist such members of the veterinary profession, their widows and orphans, who might prove to be deserving of professional benevolence. The French association numbers 940 veterinarians this year against 359 in 1897. It has a fund of $12,000.

The New York Physicians' Mutual Aid Association, incorporated in 1868, had at the beginning of this year a membership of over 1500, with a permanent fund of $40,000. It gives $1000 to a member's family at his death, and renders assistance in adversity during life. The actual expense per member for 1900 was $18.

Our Association can and ought to make a beginning that will before many years be of assistance and benefit to its members. Deeming this a subject of great importance for the future welfare of the Association, I would suggest that a committee be appointed to report immediately, so that definite action may be taken by the Association at this meeting.

The *American Veterinary Review*—that journal to which our Association a quarter of a century ago gave its support—has steadily grown in influence, and to-day is a factor in the formation of opinions among the members of our profession. At its head to-day is the same man who took the burden of nursing it into life. A few years after the publication of this paper, in 1880, THE JOURNAL OF COMPARATIVE MEDICINE AND VETERINARY ARCHIVES, so favorably known to us all, appeared. In order to succeed in any profession it is essential for us to keep in touch with the advance of the day. The press is the factor we must not overlook; it is the guide that blazes the trail to all professional progress.

In the past a great deal of our knowledge has come to us through the work of foreign schools. Has the veterinary profession of America been backward in supplying the world with new facts? I most emphatically say, No! Can any country show an equal amount of energy in dealing with contagious diseases? Can any country show such practical results in immunizing cattle by which an industry within our borders has been so revolutionized that it means untold wealth? Could this knowledge have become of such national importance without the aid of the press? Again I say, most emphatically, No!

These are facts that are of great import to us as a body; and I, fully appreciating it, trust that each and every one will benefit himself, and thereby the community of which he is a component part, by keeping in touch with the new thought as presented in our veterinary journals, remembering that added patronage increases the power of the press.

Death has made serious inroads in our ranks this past year. As our last meeting was being brought to a close the assassin's blow was struck which plunged the nation into mourning and took from us that staunch friend of the veterinarian—our beloved President of the United States, William McKinley. All nations mourned with us at the untimely end of that great and good man. The story of his life and death will forevermore be an undying example of how a citizen should live and a Christian gentleman may die. Well was it said of him: "No tenderer knight to his chosen lady ever lived. Ambitious, his sympathy made and kept friends; persuasive in his arguments, by appealing to reason and intelligence he had the confidence of all, and his support was loyal." Never were his friends embarrassed by imprudent or unguarded statements, and such was his courtesy at all times that his opponents never were offended.

John Faust, one of the oldest and most highly esteemed citizens of Poughkeepsie, New York, a member of our Association since 1884, died in the month of July, 1901. His death, from general paralysis, was indirectly the result of an injury received in a railroad accident about ten years previously.

Born in Germany in 1835, he came to this country in 1852. Having learned the cooper's trade, he started in business with two brothers, in 1859, at Poughkeepsie, N. Y. All his spare time was given to the study of our profession, and in 1875, the business partnership being dissolved, he devoted his entire time to the study and practice of veterinary medicine and surgery. His success was such that in 1881 the New York Examining Board issued a certificate to him. He served with credit as a member of the Board of Health for several years, and at the time when the question of tuberculosis was being investigated by the State he was appointed by the Governor of New York as an inspector of cattle.

He was the last member elected to our Association who by self-sacrifice had qualified himself. He prized his membership very highly, and was seldom absent from our meetings.

On November 20, 1901, Thomas F. Barron, of Baltimore, Md., died from a complication of diseases, aged fifty-nine years. Following in the footsteps of his father, a self-made man, from his early boyhood he had devoted himself to his profession. He became a member of this Association in 1887, and was a constant attendant at the meetings. A close student, always a gentleman, he was never known to speak disrespectfully of any man.

December 17, 1901, occurred the death of one whose whole life was devoted to his chosen profession. Not only his untiring energy, but his fortune, was contributed to bettering the condition and establishing the prestige of the veterinarian. To him belongs the honor of organizing the Veterinary Department of the University of Pennsylvania, and no one was better qualified to fulfil that trust. Having a liberal education and a broad knowledge of men, he was always ready to give credit when due, but he was without sympathy for an opportunist, and caustic in his denunciation of one. His friendship was to be desired, for once gained he remained ever true to that bond. This Association and others to which he belonged showed their appreciation of him by conferring on him the highest honors they were privileged to bestow. His reputation as a veterinarian was of a national character, and his services were sought and obtained not only by the Government of the United States, but by various associations of the country, to which his broad education, associated with his efforts and labor, made him so well known.

The death of Rush Shippen Huidekoper leaves a vacancy in this Association that will be difficult to fill.

In the death of Robert J. Saunders, who passed away, in his sixty-sixth year, at his home in West Roxbury, Mass., one of the original members of the United States Veterinary Medical Association was removed. A self-made man, he took up the profession of his father. Thoroughly conscientious, self-sacrificing, and studious, he commanded the respect and good-will of all who knew him. His life was spent in the city of Salem, Mass., with the exception of about one year, when on account of rheumatism he was obliged to give up active practice.

The suggestions I have offered and shall offer are made with the hope that the past harmony which has prevailed in the Association may continue. "The Romans until the fall of the empire maintained that all men were equal." "Character and talent will find their level without regard to costume." "Superiority is an attribute of character that is attained by education — practical, literary, and social."

"No member fulfils his duty to this organization who sets himself to find excuses and evasions to escape from constitutional obligations." "In times of excitement some may not have stopped to consider these, but have followed what seemed to be the current of thought. Had they fully investigated the real question and borne in mind their constitutional obligations, certainly they would have fulfilled them with alacrity."

Since the present makes the future, in the words of Daniel Webster "Let us cherish those hopes that belong to us; let us devote ourselves to those objects that are fit for our consideration and our action." "Let us raise our conceptions to the magnitude and the importance of the duties that devolve upon us. Let us preserve our Constitution, and harmony will prevail."

